# Intratumoral CRH modulates immuno-escape of ovarian cancer cells through FasL regulation

**DOI:** 10.1038/sj.bjc.6603918

**Published:** 2007-07-31

**Authors:** V Minas, A Rolaki, S N Kalantaridou, J Sidiropoulos, S Mitrou, G Petsas, U Jeschke, E A Paraskevaidis, G Fountzilas, G P Chrousos, N Pavlidis, A Makrigiannakis

**Affiliations:** 1 Laboratory of Human Reproduction, Department of Obstetrics and Gynecology, Faculty of Medicine, University of Crete, Heraklion 71003, Greece; 2 Department of Obstetrics and Gynecology, Faculty of Medicine, University of Ioannina, Ioannina 45100, Greece; 3 First Department of Obstetrics and Gynecology Ludwig-Maximilians-University of Munich, Munich 45100, Germany; 4 School of Medicine, Aristotle University of Thessaloniki, Thessaloniki, Greece; 5 First Department of Pediatrics, Athens University Medical School, Athens, Greece; 6 Department of Medical Oncology, Faculty of Medicine, University of Ioannina, Ioannina 45100, Greece

**Keywords:** CRH, Fas ligand, ovarian cancer, immune privilege, apoptosis

## Abstract

Although corticotropin-releasing hormone (CRH) and Fas ligand (FasL) have been documented in ovarian carcinoma, a clear association with tumour progression and immuno-escape has not been established. FasL plays an important role in promoting tumour cells' ability to counterattack immune cells. Here, we examined immunohistochemically the expression of CRH, CRHR1, CRHR2 and FasL in 47 human ovarian cancer cases. The ovarian cancer cell lines OvCa3 and A2780 were further used to test the hypothesis that CRH might contribute to the immune privilege of ovarian tumours, by modulating FasL expression on the cancer cells. We found that CRH, CRHR1, CRHR2 and FasL were expressed in 68.1, 70.2, 63.8 and 63.8% of the cases respectively. Positivity for CRH or FasL expression was associated with higher tumour stage. Finally, CRH increased the expression of FasL in OvCa3 and A2780 cells through CRHR1 thereby potentiated their ability to induce apoptosis of activated peripheral blood lymphocytes. Corticotropin-releasing hormone produced by human ovarian cancer might favour survival and progression of the tumour by promoting its immune privilege. These findings support the hypothesis that CRHR1 antagonists could potentially be used against ovarian cancer.

Epithelial ovarian cancer causes more deaths than any other gynaecologic cancer mainly because it is diagnosed at an advanced stage and more than half of patients have remission after surgical debulking and primary chemotherapy. Ovarian carcinoma may be recognised and attacked by the immune system. It has been reported that the presence of intratumoral T cells correlate with improved clinical outcome in advanced ovarian carcinoma (Zhang *et al*, 2003).

Peripheral corticotropin-releasing hormone (CRH) has been detected in ovarian carcinoma (Suda *et al*, 1986). Thereafter, CRH and its receptors have been localised in a number of tumours, but the role of the neuropeptide in the biology of cancer is still unclear (Ciocca *et al*, 1990; Sato *et al*, 2002; Reubi *et al*, 2003). Evidence for an anti-proliferative effect of CRH in human endometrial adenocarcinoma cells as well as in human and rat mammary cancer cells has been reported (Tjuvajev *et al*, 1998; Graziani *et al*, 2002, 2007).

The proapoptotic molecule Fas ligand (FasL) is stored in the intracellular granules of T and NK cells and can be delivered to the cell surface upon non-specific activation of these cells (Bossi and Griffiths, 1999). FasL binds to its receptor Fas and induces apoptosis of the Fas-bearing cell. FasL is also produced by non-immune cells, such as Sertoli cells in the testis, corneal endothelium and epithelium of the eye, extravillous trophoblasts (EVT) at the implantation site and several types of tumour cells (Bellgrau *et al*, 1995; Suda *et al*, 1995; Griffith *et al*, 1996; Makrigiannakis *et al*, 2001; O'Connell *et al*, 2001). In these cases, FasL attacks activated Fas-expressing immune cells, and is therefore crucial for the immune privilege of the tissues expressing it.

We have previously reported that CRH acts on EVT through CRHR1 to upregulate FasL expression and thus potentiate the ability of the cells to kill activated maternal T lymphocytes (Makrigiannakis *et al*, 2001). Corticotropin-releasing hormone has also been shown to upregulate the expression of FasL in PC12 rat pheochromocytoma cells, by activating CRHR1 (Dermitzaki *et al*, 2002).

In the present study, we examined the distribution of CRH, CRHR1, CRHR2 and FasL peptides in ovarian carcinoma and we investigated the potential role of CRH and FasL in modulating the immune defenses of ovarian cancer cells.

## MATERIALS AND METHODS

### Immunohistochemistry

The tissue material (47 cases of ovarian cancer, of which 10 were stage II, 25 were stage III and 12 were stage IV) was selected following histological review from the files of the Hellenic Cooperative Oncology Group (HeCOG). This study was approved by the Ethical Committee of the Medical School, University of Ioannina, Ioannina. For immunohistochemistry, serial sections of 4–6 *μ*m were deparaffinised in xylene and rehydrated in graded alcohol to TBS (50 mM Tris, 150 mM NaCl, pH 7.4). The latter was used for washes between steps. The slides were microwaved for 4 × 5 min in 10 mM citrate, pH 6.0. After cooling down for 20 min, the slides were blocked for 30 min at room temperature (rt) with normal serum (rabbit IgG, ABC Kit, Vector Laboratories, Burlingame, CA, USA), diluted 1 : 20 and then incubated with one of the primary antibodies (CRH antibody was obtained from Phoenix Pharmaceuticals, Burlingame, CA, USA and Corticotropin-releasing hormones like CRHR1, CRHR2 and FasL antibodies were obtained from Santa Cruz, CA, USA) overnight in a dilution 1/100 in TBS plus 2% bovine serum albumin (BSA). The slides were then incubated with the appropriate biotinylated secondary antibody (BioGenex, San Ramon, CA, USA) for 20 min at rt. Finally, the slides were incubated for 20 min with streptavidin-conjugated horseradish peroxidase (BioGenex) and the peptides were localised using 3,3′-diaminobenzidine tetrahydrochloride as chromogen (BioGenex). Tissues were counterstained with haematoxylin (BioGenex), mounted with coverslips and photographed with a Nikon digital camera, Tokyo, Japan under an Olympus microscope, Olympus Optical Co, Tokyo, Japan.

The intensity and distribution patterns of the staining reaction were evaluated by two blinded, independent observers, including a gynaecological pathologist, using the semiquantitative immunoreactive score (IRS), as described previously (Remmele *et al*, 1986). The IRS was calculated by multiplication of optical staining intensity (graded as 0=no, 1=weak, 2=moderate and 3=strong staining) and the percentage of positive-stained cells (0=no staining, 1=<10% of the cells, 2=11–50% of the cells, 3=51–80% of the cells and 4=>81% of the cells) and without having knowledge of the pathological evaluation, the diagnosis or the standard performed haematoxylin reaction of each specimen.

### Cell lines

The OvCa3 and A2780 ovarian cancer cell lines were used for the *in vitro* experiments. The cells were kept in DMEM (GibcoBRL, Gaithersburg, MD, USA) supplemented with 10% FCS (GibcoBRL) and antibiotics. The cells were kept in incubators at 37°C and 5% CO_2_.

### RT–PCR

Total RNA was extracted from cultures of OvCa3 and A2780 cells using the RNA extraction II kit (Molecular Probes, Carlsbad, CA, USA) according to the manufacturer's instructions. Five micrograms of total RNA were reverse-transcripted using a reverse transcription kit (Invitrogen Life Technologies, Carlsbad, CA, USA). An aliquot of 1/20th of the resulting cDNA was used for PCR amplification using the appropriate kit according to the manufacturer's instructions (Invitrogen Life Technologies).

For CRH, primers were: sense 5′-CAACTTTTTCCGCG TGTTGCT-3′, antisense 5′-ATGGCATAAGAGCAGCGCTAT-3′, which amplified a fragment of 248 bp. The PCR conditions consisted of an initial denaturing step of 98°C for 5 min, followed by 40 cycles (95°C for 1 min, 60°C for 1 min, 72°C for 1 min) and a final step of 72°C for 7 min. For CRHR1, primers were: sense 5′-AAGGTGC ACTACCATGTCGCA-3′, antisense 5′-GGTCATGAGGATGCGACG-3′ which amplified a fragment of 272 bp. The PCR conditions consisted of an initial denaturing step of 72°C for 1 min, followed by 40 cycles (96°C for 5 min, 96°C for 40 s and 61°C for 45 s) and a final step of 72°C for 5 min. For CRHR2, primers were: sense 5′-GACGCGGCACTGCTCCACAG-3′, antisense 5′-GCATTCCGGGTC GTGTTGT-3′, which amplified a fragment of 233 bp. The PCR conditions consisted of an initial denaturing step of 98°C for 5 min, followed by 40 cycles (95°C for 45 s, 62°C for 45 s and 72°C for 45 s) and a final step of 72°C for 1.30 min. For FasL, primers were: sense 5′-ACACCTATGGAATTGTCCTGC-3′, antisense 5′-GACCAGAGAGAGCTCAGATACG-3′, which amplified a fragment of 311 bp. The PCR conditions consisted of an initial denaturing step of 94°C for 3 min, followed by 35 cycles (92°C for 10 s, 55°C for 30 s and 72°C for 60 s) and a final step of 72°C for 7 min. A 10 *μ*l aliquot of amplified product was electrophoresed through a 2% agarose gel, stained with ethidium bromide, and photographed under ultraviolet-transillumination. Total placental RNA was used as positive control. Negative controls included substitution of reverse-transcriptase with water (no-RT).

### Indirect immunofluorescence

OvCa3 and A2780 cells grown on coverslips were washed in TBS, and then fixed in 100% methanol at −20°C for 5 min. Cells were incubated with 10% normal bovine serum (30 min at rt) and then with one of the primary antibodies overnight at 4°C. Primary antibodies against CRH (Phoenix Pharmaceuticals), CRHR1, CRHR2 and FasL (all from Santa Cruz) were diluted 1 : 100 plus 2% BSA. The appropriate FITC- or TRITC-conjugated secondary antibody (Chemicon, Temecula, CA, USA) diluted 1 : 100 in TBS, was added and the cells were incubated for 1 h at rt. DAPI (Molecular Probes) was used for visualisation of the nuclei and coverslips were mounted on glass slides. Negative controls were obtained by omission of primary antibodies. The cells were photographed with a Leica digital camera under an Olympus microscope.

### Western blot analysis

For analysis of cellular proteins, cells were lysed using lysis buffer (Chemicon). Lysates were boiled in sample buffer for 10 min at 96°C. Samples, containing 50 *μ*g protein were separated by 12% SDS–PAGE gel, and then transferred onto a nitrocellular membrane (Schleicher & Schuel, Sigma-Aldrich, St Louis, MO, USA). Membranes were then blocked for 1 h at rt using 5% milk powder in TBS. Each membrane was incubated overnight at 4°C with primary anti-FasL antibody diluted 1 : 400 and then incubated at rt for 1 h with the appropriate peroxidase-conjugated secondary antibody (Chemicon). Detection of proteins was carried out using enhanced chemiluminescence (NEN Life Sciences, Boston, Massachusetts, USA). To normalise for protein content, the blots were stripped in stripping buffer (62.5 mM Tris-HCl, pH 6.7, 2% SDS, 100 mM *β*-mercaptoethanol) and stained with anti-actin antibody (Chemicon). The concentration of FasL protein in each lysate was normalised against actin. The intensity of the bands was quantified using the ImageJ Imaging System, NIH, Bethesda, MD, USA.

### Peripheral blood lymphocyte isolation and activation

Normal donor blood was diluted two-fold and peripheral blood mononuclear cells were purified by Ficoll-Paque (Sigma, St Louis, MO, USA) density-gradient centrifugation. The cells at the interface were collected in culture in complete culture medium (RPMI-1640, 10% FCS, 100 U ml^−1^ penicillin, and 50 g ml^−1^ gentamicin, all from GibcoBRL), and incubated for 2 h at 37°C in an atmosphere of 5% CO_2_ to allow adherent cells to attach to the plastic. The supernatant containing peripheral blood lymphocytes (PBL) was collected. To induce Fas expression, PBL were activated by culturing in complete RPMI-1640 medium supplemented with 50 U ml^−1^ interleukin-2 for 3 days (Meng *et al*, 2004).

### Apoptosis assays

OvCa3 and A2780 cells were grown in six-well plates in their respective media with or without the presence of 100 nM CRH (Tocris)±1 *μ*M antalarmin (gift from GP Chrousos). After 48 h, the media were changed to RPMI with 10% FCS and 10^5^ activated PBL were added to the wells for each treatment, which resulted in target : effector cell ratios of approximately 1 : 20. Treatments in co-cultures included addition of 10 nM CRH±antalarmin, and addition of 10 nM CRH±2 *μ*g of an anti-Fas blocking antibody according to the manufacturer's instructions (SM1/23, ALEXIS Biotechnologies, Lausen, Switzerland). When the anti-Fas antibody was used, PBL were treated with it for 1 h before co-cultures. After 24 h in co-culture, the wells were gently rinsed to recover the non-adherent PBL. The latter were either cytospined (for TUNEL and poly-caspase activation detection assay) or lysed (for caspase-8 colorimetric assay). Apoptosis was detected (i) by TUNEL assay (terminal deoxynucleotidyl transferase-mediated dUTP nick end labelling) (Boehringer-Mannheim) (ii) by fluorescence staining of activated caspases using a poly-caspase activation-detection kit (the kit stains activated caspase-1, -3, -4, -5, -6, -7, -8 and -9) (Molecular Probes), and (iii) by a caspase-8 colorimetric assay (R&D systems, Minneapolis, MN, USA). All three kits are commercially available and were used according to manufacturers' instructions. In assays (i) and (ii), DAPI was used for the visualisation of nuclei and apoptotic cells were visualised under an Olympus fluorescence microscope and photographed with a Leica camera. Five random × 20 microscopic fields per slide were photographed and the number of positively fluoresced cells and the total number of cells were counted. Each slide was independently evaluated by two investigators. For controls, PBL were additionally subjected to the three apoptosis assays after cultured alone for 24 or 48 h with 10 or 100 nM CRH.

### Cell growth analysis

Cell proliferation was evaluated by MTT (3-[4,5-dimethylthiazol-2-yl]-2,5-diphenyl tetrazolium bromide) assay (Sigma) and cell counting. For MTT assay, OvCa3 or A2780 cells were seeded in 96-well plates and treated with 10 or 100 nM CRH, or medium only for 24, 48 or 72 h (five wells per condition, 10^4^ cells per well). Fresh CRH was added to the culture every 24 h. At the end of the incubation period, the MTT assay was performed according to the manufacturer's instructions. Plates were read at 620 nm with an ELISA microplate reader (BioRad, Hercules, CA, USA). For cell counting, cells were grown in flasks (10^5^ cells per flask) and same treatments were performed. Cells numbers were estimated (using Trypan blue exclusion) at 24, 48 and 72 h.

### Statistics

Statistical analysis was performed using the non-parametrical Mann–Whitney *U*-test and the unpaired two-tailed Student's *t*-test (assuming equal variances). Only *P*<0.05 was considered significant.

## RESULTS

### Human ovarian cancer produces the CRH, CRHR1, CRHR2 and FasL peptides

**Figure 1:**
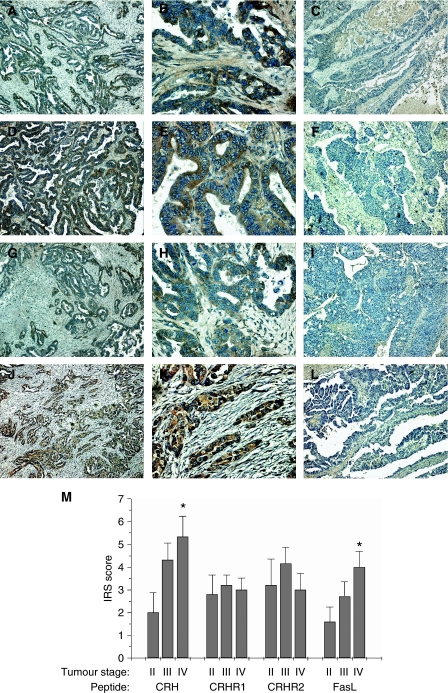
Immunohistochemical expression of CRH, CRHR1, CRHR2 and FasL in ovarian cancer tissue. (**A**–**L**) Representative photos of cases, which expressed or did not express the investigated peptides. CRH was immunolocalised in tumour cells in 68.1% of the cases examined (**A**, **B**), whereas the rest (31.9%) did not express the peptide (**C**). CRHR1 was expressed in 70.2% of cases (**D**, **E**) and absent from 29.8% (**F**). CRHR2 was expressed in 63.8% (**G**, **H**) of cases and absent from 36.2% (**I**). Finally, 63.8% of the cases were positive for FasL (**J**, **K**) and 36.2% were negative (**L**). Lens × 10 (**A**, **C**, **D**, **F**, **G**, **I**, **J**, **L**), × 40 (**B**, **E**, **H**, **K**). (**M**) Staining intensity was determined by the semiquantitative immunohistochemical IRS. There was a statistically significant increase in CRH and FasL expression between stage II and stage IV tumours. Data represent mean±s.e. (^*^P<0.05).

Immunohistochemistry on 47 cases of human ovarian cancer revealed that human ovarian tumour cells produce the investigated peptides *in situ*. Overall, the percentages of tumours which were found to express one of the peptides CRH, CRHR1, CRHR2 and FasL were 68.1, 70.2, 63.8 and 63.8%, respectively (examples of tissues positive or negative for one of the peptides are shown in Figure 1). Tumour advancement, as assessed by increasing tumour stage, was associated with significantly increased immunohistochemical positivity for CRH and FasL (*P*<0.05 between stage II and stage IV tumours) (Table 1). This correlation was further confirmed by IRS scoring. In this case, tumour advancement was positively correlated with the comparatively estimated amount of CRH or FasL produced by tumour cells (Figure 1) (*P*<0.05 between stage II and stage IV tumours). Furthermore, tumour advancement was positively correlated with the percentage of tumours simultaneously expressing CRH, CRHR1 and FasL. These three peptides were concurrently expressed by 30% of stage II, 48% of stage III and 66.7% of stage IV tumours, whereas the overall percentage of tissues positive for all three peptides was 48.9%. This difference however, was not statistically significant (Table 1).

No significant differences in the expression of the examined molecules were detected between groups of tumours with different histological grade or type (data not shown).

### OvCa3 cells express CRH, CRHR1, FasL and A2780 express CRH, CRHR1, CRHR2, FasL mRNAs and peptides

**Figure 2:**
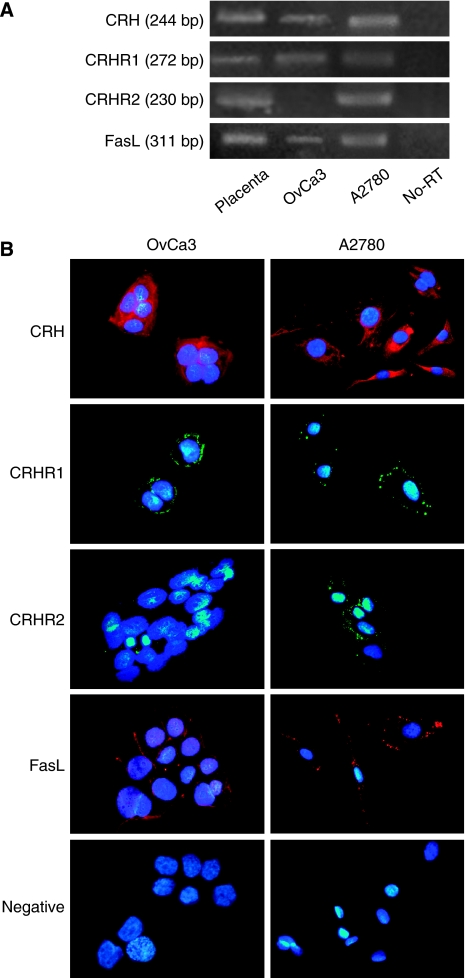
Expression of CRH, CRHR1, CRHR2 and FasL mRNAs and peptides in OvCa3 and A2780 cells. (**A**) RT–PCR was performed in total mRNA extracted from OvCa3 and A2780 cells. Amplification of cDNA generated bands of expected sizes, representing the expression of the *CRH* (244 bp), *CRHR1* (272 bp), *CRHR2* (230 bp) and *FasL* (311 bp) genes, with the exception of *CRHR2*, which was not expressed in OvCa3 cells. Total placental mRNA was used as positive control. Omission of reverse transcriptase served as negative control to exclude material or genomic DNA contamination (no-RT). (**B**) Immunofluorescence experiments revealed the expression of CRH, CRHR1, CRHR2 and FasL peptides, again with the exception of CRHR2 which was not detected in OvCa3 cells. CRH was detected in the cytoplasm whereas CRHR1 and FasL were localised mainly on the cell membrane. For negative controls, primary antibodies were omitted. Lens × 40.

RT–PCR was performed in total mRNA extracted from OvCa3 and A2780 cells, with the use of appropriate primers. Amplification of cDNA generated bands of expected sizes, representing the expression of the *CRH*, *CRHR1*, *CRHR2 and FasL* genes, with the exception of *CRHR2*, which was not expressed in OvCa3 cells (Figure 2A). Total placental mRNA was used as positive control, since placenta expresses mRNAs of the examined genes. Omission of reverse transcriptase served as negative control to exclude material or genomic DNA contamination (no-RT). Immunofluorescence experiments revealed the expression of the respective peptides in both cell lines, again with the exception of CRHR2, which was absent from OvCa3 cells (Figure 2B). Corticotropin-releasing hormone was detected in the cytoplasm whereas CRHR1, CRHR2 and FasL were mainly localised on the cell membranes. Negative controls were obtained after omission of primary antibodies.

### CRH upregulates the expression of FasL in OvCa3 and A2780 cells through CRHR1

**Figure 3:**
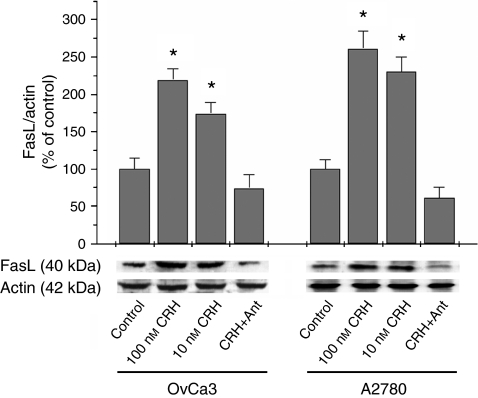
Modulation of FasL expression in OvCa3 and A2780 cells by CRH. OvCa3 and A2780 cells in culture were incubated for 48 h with 10 or 100 nM of CRH. Ten and 100 nM of CRH significantly increased FasL expression in OvCa3 and A2780 cells. This effect was mediated by CRHR1, since the addition of 10-fold higher concentration of the specific antagonist antalarmin (Ant), completely reversed it. Data represent mean±s.e. (*n*=4) (^*^P<0.05).

OvCa3 and A2780 cells were incubated with CRH with or without the presence of antalarmin, a CRHR1-specific antagonist, at 10-fold higher concentration. After 48 h incubation with 10 or 100 nM CRH, expression of FasL in OvCa3 cells was increased 1.7- and 2.2-fold, respectively compared to control. The respective figures for A2780 cells were 2.3- and 2.6-fold. This effect was specifically mediated by CRHR1, since the addition of 1 *μ*M antalarmin completely reversed it. The experiment was performed four times (Figure 3) (*P*<0.05).

### CRH potentiates the ability of OvCa3 and A2780 cells to induce Fas-mediated apoptosis of activated lymphocytes

**Figure 4:**
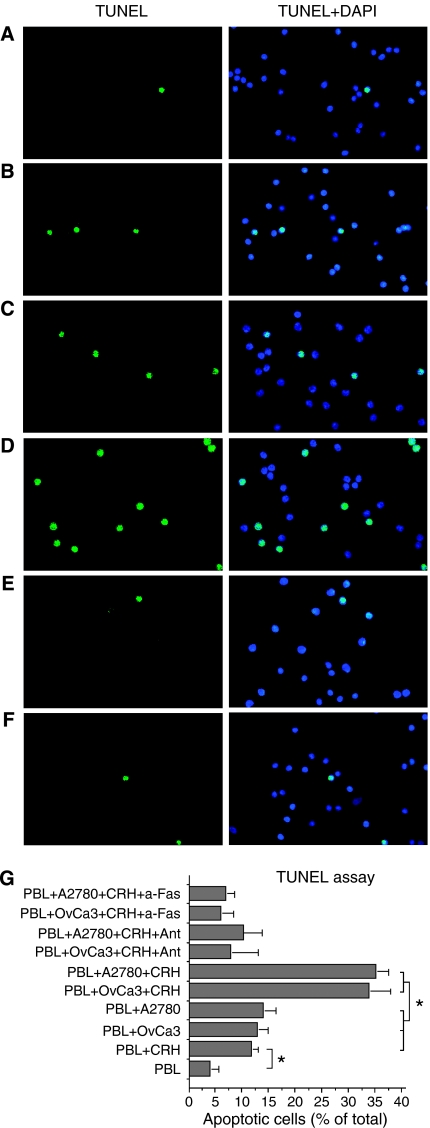
TUNEL assay in activated lymphocytes after co-culturing with OvCa3 or A2780 cells. (**A**–**F**) Representative TUNEL assay photos of PBL after 24 h culturing in various conditions. Activated PBL cultured alone for 24 h showed only limited apoptosis (**A**). In the presence of 10 nM CRH, PBL apoptosis was moderately increased (**B**). Similarly, moderate levels of PBL apoptosis occurred after co-culturing with OvCa3 or A2780 cells (**C**). Activated PBL cultured with OvCa3 or A2780 cells primed with 100 nM CRH for 48 h, then with the continuous presence of 10 nM CRH for 24 h, showed high levels of apoptosis (**D**). Activated PBL cultured with OvCa3 or A2780 cells primed with 100 nM CRH+1 *μ*M antalarmin for 48 h, then with 10 nM CRH+100 nM antalarmin for 24 h, reverted the effects of CRH (**E**). Similarly, addition of an anti-Fas blocking antibody in co-cultures with CRH-treated cells, reverted the effects of CRH (**F**). (**G**) Quantitation of results. Data represent mean±s.e. (*n*=4) (^*^P<0.05).

**Figure 5:**
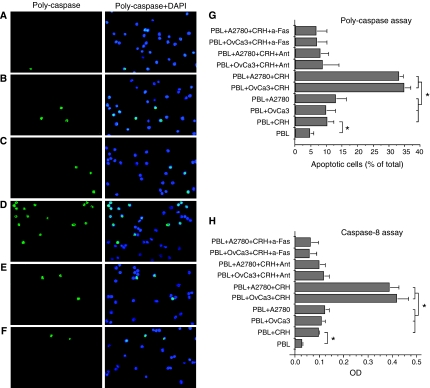
Poly-caspase assay and caspase-8 colorimetric assay in activated lymphocytes after co-culturing with OvCa3 or A2780 cells. (**A**–**F**) Representative poly-caspase assay photos of PBL after 24 h culturing in various conditions. Poly-caspase assay confirmed the results obtained from TUNEL assay, as described in Figure 4. (**G**) Quantitation of results. Data represent mean±s.e. (*n*=4) (^*^P<0.05). (**H**) Caspase-8 enzymatic activity in activated PBL cultured as described above. Results were similar to those described in Figures 4G and 5G. Data represent mean±s.e. (expressed as O.D. units, optical density) (*n*=4) (^*^P<0.05).

Cytospin preparations of activated PBL co-cultured with OvCa3 or A2780 cells in the presence or absence of CRH±antalarmin, were subjected to TUNEL and poly-caspase assays and examined under fluorescence microscopy. When activated PBL were cultured for 24 h with FasL-expressing OvCa3 or A2780 cells in the absence of CRH, a small percentage of the lymphocytes underwent apoptosis (13±2% in TUNEL and 10±3% in poly-caspase assay for co-cultures with OvCa3, 14±2 and 13±3%, respectively for co-cultures with A2780) (Figure 4C, G and Figure 5C, G). However, when activated PBL were cultured with OvCa3 or A2780 cells, which were pre-incubated for 48 h with 100 nM CRH, in the continuous presence of 10 nM CRH, significantly higher percentages of the lymphocytes underwent apoptosis (34±4% in TUNEL and 35±2% in poly-caspase assay for co-cultures with OvCa3, 35±2 and 33±1%, respectively for co-cultures with A2780) (Figures 4D, G and 5D, G, respectively) (*P*<0.05). This effect was mediated by CRHR1, since the addition of 1 *μ*M antalarmin reduced apoptosis to control amounts (Figures 4E, G and 5E, G). To establish a role for FasL in PBL apoptosis, an anti-Fas blocking antibody was employed. Addition of 2 *μ*g of the antibody, blocked the effect of CRH-treated ovarian cancer cells on PBL apoptosis (6±2% apoptotic PBL in TUNEL and 7±3% in poly-caspase assay for co-cultures with OvCa3, 7±1% in TUNEL and 7±3% in poly-caspase for co-cultures with A2780) (Figures 4F, G and 5F, G). Finally, to exclude the possibility of a potent cytotoxic effect of CRH against activated PBL, the latter were cultured alone±10 or 100 nM CRH for 24 or 48 h. Basic levels of PBL apoptosis after 24 h in culture were 4±1.5% in TUNEL and 5±1% in poly-caspase assay (Figures 4A, G and 5A, G). Addition of 10 nM CRH increased apoptosis of PBL after 24 h in culture (12±1% in TUNEL and 10.5±2% in poly-caspase assay) (Figures 4B, G and 5B, G) (*P*<0.05). Results obtained with different CRH concentrations (10 or 100 nM) and at different time intervals (24 or 48 h) did not differ significantly (data not shown). In all cases, apoptosis was significantly lower than in co-cultures with CRH-treated ovarian cancer cells (*P*<0.05). All experiments were performed four times (*P*<0.05).

Following the same treatments, activated PBL were lysed and subjected to caspase-8 colorimetric assay. Results were similar with those from the two aforementioned assays (Figure 5H). The experiment was performed four times (*P*<0.05).

### CRH does not affect growth of OvCa3 or A2780 cells

**Figure 6:**
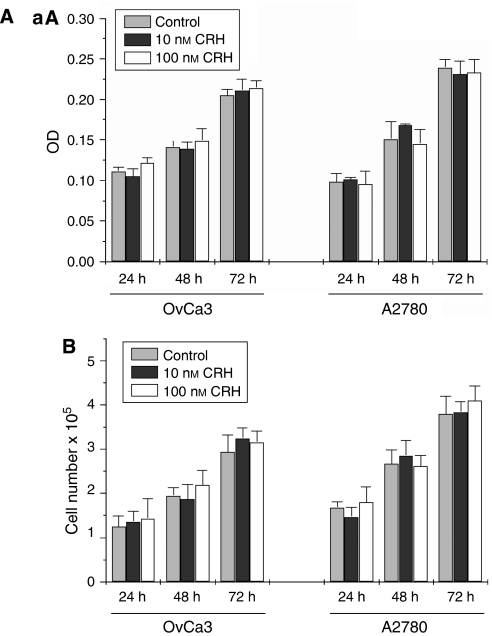
Cell growth analysis in CRH-treated OvCa3 and A2780 cells. (**A**) MTT assay in CRH-treated ovarian cancer cells. Corticotropin-releasing hormone had no effect in growth of OvCa3 or A2780 cells. Absorbance (expressed as O.D. units, optical density) is proportional to numbers of living cells. Data represent mean±s.e. (*n*=4). (**B**) Growth of ovarian cancer cells was additionally estimated by counting living cell numbers (Trypan blue exclusion). Corticotropin-releasing hormone did not alter cell numbers significantly. Data represent mean±s.e. (*n*=4).

To investigate the potential effect of CRH on ovarian cancer cell growth, MTT assay and cell counting were performed. Corticotropin-releasing hormone at 10 or 100 nM had no effect on cell growth after 24, 48 or 72 h of treatment compared to medium alone (control) (Figure 6). All experiments were performed four times.

## DISCUSSION

Our study indicates that intratumoral expression of CRH and FasL is associated with advanced tumour stage in ovarian cancer. The expression of the two peptides was significantly higher in stage IV compared to stage II tumours. The percentage of tumours simultaneously expressing CRH, CRHR1 and FasL was higher in stage IV compared to stage II tumours, although this difference was not statistically significant. In addition, we present data that might offer an explanation on the association of intratumoral CRH with tumour advance. We found that CRH upregulates the expression of FasL in two human ovarian cancer cell lines (OvCa3 and A2780), thereby potentiating their ability to induce Fas-mediated apoptosis of activated PBL. Finally, CRH had no effect on growth of OvCa3 or A2780 cells.

Corticotropin-releasing hormone is the principal regulator of the stress response in mammals (Bale and Vale, 2004). Outside the central nervous system, CRH is produced in several peripheral tissues, where it acts as an autocrine and/or paracrine regulator of various cellular functions (Boorse and Denver, 2006). Corticotropin-releasing hormone mRNA and peptide have been detected in both human and rat normal ovaries. More specifically, immunoreactive CRH (irCRH) and its binding sites have been localised in thecal and stromal cells of rat and human ovaries and in human follicular fluid (Mastorakos *et al*, 1993, 1994). Type 1 (CRHR1) and type 2 (CRHR2) CRH receptor mRNAs have also been detected in corpus luteum (Muramatsu *et al*, 2001). It is currently suggested that the role of the locally expressed CRH system in normal ovary, is to regulate—more likely suppress—ovarian steroidogenesis (Calogero *et al*, 1996; Ghizzoni *et al*, 1997; Erden *et al*, 1998).

Corticotropin-releasing hormone and its receptors have been detected in a number of primary human tumours. Corticotropin-releasing hormone is expressed in melanoma (Sato *et al*, 2002), pheochromocytoma, ovarian cancer (Suda *et al*, 1986) and breast cancer (Ciocca *et al*, 1990). Corticotropin-releasing hormone receptors have been detected in melanoma (Funasaka *et al*, 1999), in pancreatic and central and peripheral nervous system tumours (Reubi *et al*, 2003). The present study confirms the expression of CRH and reports for the first time the expression of CRH receptors in ovarian cancer *in situ*. Therefore, CRH may act in a paracrine and/or autocrine way to modulate various functions of ovarian tumour cells. Furthermore, this study associates the expression of CRH with tumour advancement to higher stages, thus proposing that the tumour might benefit from CRH secretion.

In addition to CRH, stage IV tumours upregulated FasL. The latter is currently considered an important part of tumours' defences against immune attack. In parallel to FasL expression, cancer cells often display resistance to Fas-mediated apoptosis and therefore ‘the Fas counterattack’ concept was introduced to describe the modulation of the Fas–FasL system by tumour cells to favour their immune privilege (O'Connell *et al*, 1999).

The present results are in agreement with previous studies reporting the expression of FasL in different tumours and the association of the molecule with the disease's clinical course. FasL has been detected in several tumours of varying origin, including colon (Okada *et al*, 2000), breast (Mottolese *et al*, 2000; Reimer *et al*, 2000), liver (Ito *et al*, 2000), gastric (Zheng *et al*, 2003) and lung carcinoma (Niehans *et al*, 1997; Volm and Koomagi, 2000), astrocytoma (Saas *et al*, 1997), esophageal (Shibakita *et al*, 1999), renal (Kim *et al*, 2000) and ovarian tumours (Munakata *et al*, 2000; van Haaften-Day *et al*, 2003). Most of the aforementioned reports showed that disease progression was associated with progressively increased expression of FasL. In some types of the examined tumours, including the ovarian, intratumoral lymphocytes showed either increased apoptosis or decreased density within cases which expressed FasL (O'Connell *et al*, 1999).

An elaborate study by Zhang *et al* (2003) further confirmed and revealed the significance of the intensity of immune attack to the progression of ovarian cancer. The authors concluded that the presence of tumour-infiltrating T cells in ovarian carcinoma cases correlates with improved clinical outcome. Reduced immune response against the tumour or increased tumour counterattack, as evidenced by the absence of intratumoral T cells in islets, was associated with low 5-year overall survival rate (Zhang *et al*, 2003). In our study, insufficient clinical data and restricted number of cases did not allow association of the immunohistochemical results with clinical outcome. Nevertheless, a significant correlation between FasL expression and advanced tumour stage is reported.

Although no alteration was observed in the expression of CRHRs among the three stage-groups of tumours, more stage IV than stage II tumours simultaneously expressed the CRH, CRHR1 and FasL peptides. This difference was not statistically significant due to the small number of cases. Nevertheless, we assumed that there could be a functional interrelation between the three peptides. To explore this hypothesis, the ovarian cancer cell lines OvCa3 and A2780 were employed. OvCa3 cells were found to express CRH, CRHR1 and FasL, but not CRHR2 transcripts and peptides, whereas A2780 expressed all of the above transcripts and peptides. In both cell lines, CRH induced FasL through CRHR1. This was in accord with previous reports on rat pheochromocytoma PC12 cancer cells (Dermitzaki *et al*, 2002), and on EVT, a particular cell type sharing similarities with cancer cells (Makrigiannakis *et al*, 2001). This effect of CRH was further found to have a role in mediating an attack of OvCa3 and A2780 cells against activated PBL, again in agreement with the case of EVT (Makrigiannakis *et al*, 2001). When PBL were placed in co-cultures with OvCa3 or A2780 cells in the presence of CRH, the number of PBL, which underwent apoptosis significantly increased compared to co-cultures without CRH, or with CRH and antalarmin. We suggest that apoptosis of PBL was mainly owed to Fas–FasL interaction for the following reasons: Addition of an anti-Fas blocking antibody reverted the effect of CRH-treated ovarian cancer cells, by significantly reducing the numbers of apoptotic PBL. Furthermore, CRH alone induced low levels of PBL apoptosis, comparable to those measured in co-cultures with ovarian cancer cells without any additives. Finally, in all conditions, the enzymatic activity of caspase-8 (a key component of the Fas–FasL apoptotic pathway) in PBL lysates was in accordance with levels of PBL apoptosis.

The ability of ovarian cancer cells to induce Fas-mediated apoptosis to lymphocytes has been previously reported by a number of studies using different experimental approaches (Rabinowich *et al*, 1998; Abrahams *et al*, 2003; Meng *et al*, 2004). In particular, Meng *et al* (2004) found that lysophosphatidic acid stimulates the expression of FasL in OvCa3 cells. Similarly to the present study, the authors showed that stimulated tumour cells induced apoptosis of isolated T lymphocytes more effectively than their unstimulated controls (Meng *et al*, 2004). Additional factors, which regulate FasL in tumour cells, include oestrogen and the anti-oestrogen factor tamoxifen tested on human breast cancer cells (Mor *et al*, 2000), and the bacterial polysaccharide exotoxin CM101 tested on murine melanoma cells (Yakes *et al*, 2000). The results of the present study suggest that tumour-produced CRH might be an autocrine–paracrine factor, which together with others, modulates the expression of FasL in ovarian cancer. Since a large proportion of ovarian tumours were found to express CRHR1 (70.2%), secretion of CRH might considerably improve tumour's resistance to immune attack and thus favour its survival and progression. These observations are of particular importance, since growth assays failed to reveal an anti-proliferative effect of CRH on ovarian cancer cells, which is in contrast with what has been reported on human endometrial and breast cancer cells (Graziani *et al*, 2002, 2007). Of note, CRH-stimulated angiogenesis and promoted tumour growth *in vivo* in a model of mice inoculated with human epithelial tumour cells (Arbiser *et al*, 1999). Thus, the effect of CRH on tumour growth appears to be tissue-specific and the final result *in vivo* might differ from the observed effects *in vitro* due to the contribution of several factors.

In their study, Reubi *et al* (2003) detected CRH receptors in specific tumours and expressed the very promising hypothesis that these receptors might be used as targets for long-term CRH therapy. However, our lack of knowledge on the role of CRH in cancer biology, limits our leeway for testing CRH antagonists against specific and vital molecular functions of the tumour. Non-peptidic CRHR1 antagonists have been tested in experimental animals. It has been shown that chronic use of CRHR1 antagonists in rats has no serious adverse effects (Grammatopoulos and Chrousos, 2002). It is tempting to speculate that in advanced stage ovarian carcinoma, chronic use of a CRHR1 antagonist could reduce tumour immune defence mechanisms by downregulating intratumoral FasL expression. As a result, cancer cells would be recognised and attacked by the immune system. In addition, the present results suggest that use of such substances would be unlikely to directly affect the proliferation rate of the tumour cells. Therefore, the observations reported here introduce novel insight into how such molecules could be used against cancer. This hypothesis is currently under investigation by our research group.

In summary, we found that CRH, its receptors CRHR1 and CRHR2, and the proapoptotic molecule FasL are produced by human ovarian tumour cells *in situ*. Expression of CRH and FasL were positively associated with higher tumour stage. We propose that tumour CRH acts in a paracrine–autocrine way to favour survival and progression of the tumour by promoting its immune privilege, whereas it has no direct effect on tumour cells growth rate. Indeed, CRH acted on the ovarian cancer cells OvCa3 and A2780, through CRHR1, to upregulate the expression of FasL and thus potentiate the ability of the cells to induce Fas-mediated apoptosis of activated PBL. Our study sets the initiative for the consideration of CRHR1 antagonists in an effort to reduce tumour immune defenses.

## Figures and Tables

**Table 1:** Percentages of ovarian cancer tumours expressing CRH, CRHR1, CRHR2 or FasL peptides, sorted by tumour stage

**Peptide(s)**	**Tumour stage**	**Positive cases**	**Negative cases**
CRH	II	40% (4/10)	60% (6/10)
	III	72% (18/25)	28% (7/25)
	IV	83.3% (10/12)^a^	16.7% (2/12)
CRHR1	II	60% (6/10)	40% (4/10)
	III	72% (18/25)	28% (7/25)
	IV	75% (9/12)	25% (3/12)
CRHR2	II	50% (5/10)	50% (5/10)
	III	68% (17/25)	32% (8/25)
	IV	66.7% (8/12)	33.3% (4/12)
FasL	II	40% (4/10)	60% (6/10)
	III	64% (16/25)	36% (9/25)
	IV	83.3% (10/12)^a^	16.7% (2/12)
CRH/CRHR1/FasL	II	30% (3/10)	70% (7/10)
	III	48% (12/25)	52% (13/25)
	IV	66.7% (8/12)	33.3% (4/12)

a Percentage of stage IV tumours expressing CRH or FasL was significantly higher than that of stage II tumours (*P*<0.05).

## References

[bib1] Abrahams VM, Straszewski SL, Kamsteeg M, Hanczaruk B, Schwartz PE, Rutherford TJ, Mor G (2003) Epithelial ovarian cancer cells secrete functional Fas ligand. Cancer Res 63: 5573–5581 14500397

[bib2] Arbiser JL, Karalis K, Viswanathan A, Koike C, Anand-Apte B, Flynn E, Zetter B, Majzoub JA (1999) Corticotropin-releasing hormone stimulates angiogenesis and epithelial tumor growth in the skin. J Invest Dermatol 113: 838–842 10571742 10.1046/j.1523-1747.1999.00760.x

[bib3] Bale TL, Vale WW (2004) CRF and CRF receptors: role in stress responsivity and other behaviors. Annu Rev Pharmacol Toxicol 44: 525–557 14744257 10.1146/annurev.pharmtox.44.101802.121410

[bib4] Bellgrau D, Gold D, Selawry H, Moore J, Franzusoff A, Duke RC (1995) A role for CD95 ligand in preventing graft rejection. Nature 377: 630–632 7566174 10.1038/377630a0

[bib5] Boorse GC, Denver RJ (2006) Widespread tissue distribution and diverse functions of corticotropin-releasing factor and related peptides. Gen Comp Endocrinol 146: 9–18 16413023 10.1016/j.ygcen.2005.11.014

[bib6] Bossi G, Griffiths GM (1999) Degranulation plays an essential part in regulating cell surface expression of Fas ligand in T cells and natural killer cells. Nat Med 5: 90–96 9883845 10.1038/4779

[bib7] Calogero AE, Burrello N, Negri-Cesi P, Papale L, Palumbo MA, Cianci A, Sanfilippo S, D'Agata R (1996) Effects of corticotropin-releasing hormone on ovarian estrogen production *in vitro*. Endocrinology 137: 4161–4166 8828472 10.1210/endo.137.10.8828472

[bib8] Ciocca DR, Puy LA, Fasoli LC, Tello O, Aznar JC, Gago FE, Papa SI, Sonego R (1990) Corticotropin-releasing hormone, luteinizing hormone-releasing hormone, growth hormone-releasing hormone, and somatostatin-like immunoreactivities in biopsies from breast cancer patients. Breast Cancer Res Treat 15: 175–184 1973621 10.1007/BF01806354

[bib9] Dermitzaki E, Tsatsanis C, Gravanis A, Margioris AN (2002) Corticotropin-releasing hormone induces Fas ligand production and apoptosis in PC12 cells via activation of p38 mitogen-activated protein kinase. J Biol Chem 277: 12280–12287 11790788 10.1074/jbc.M111236200

[bib10] Erden HF, Zwain IH, Asakura H, Yen SS (1998) Corticotropin-releasing factor inhibits luteinizing hormone-stimulated P450c17 gene expression and androgen production by isolated thecal cells of human ovarian follicles. J Clin Endocrinol Metab 83: 448–452 9467556 10.1210/jcem.83.2.4546

[bib11] Funasaka Y, Sato H, Chakraborty AK, Ohashi A, Chrousos GP, Ichihashi M (1999) Expression of proopiomelanocortin, corticotropin-releasing hormone (CRH), and CRH receptor in melanoma cells, nevus cells, and normal human melanocytes. J Investig Dermatol Symp Proc 4: 105–109 10.1038/sj.jidsp.5640192 10536983

[bib12] Ghizzoni L, Mastorakos G, Vottero A, Barreca A, Furlini M, Cesarone A, Ferrari B, Chrousos GP, Bernasconi S (1997) Corticotropin-releasing hormone (CRH) inhibits steroid biosynthesis by cultured human granulosa-lutein cells in a CRH and interleukin-1 receptor-mediated fashion. Endocrinology 138: 4806–4811 9348209 10.1210/endo.138.11.5474

[bib13] Grammatopoulos DK, Chrousos GP (2002) Functional characteristics of CRH receptors and potential clinical applications of CRH-receptor antagonists. Trends Endocrinol Metab 13: 436–444 12431840 10.1016/s1043-2760(02)00670-7

[bib14] Graziani G, Tentori L, Muzi A, Vergati M, Tringali G, Pozzoli G, Navarra P (2007) Evidence that corticotropin-releasing hormone inhibits cell growth of human breast cancer cells via the activation of CRH-R1 receptor subtype. Mol Cell Endocrinol 264: 44–49 17097220 10.1016/j.mce.2006.10.006

[bib15] Graziani G, Tentori L, Portarena I, Barbarino M, Tringali G, Pozzoli G, Navarra P (2002) CRH inhibits cell growth of human endometrial adenocarcinoma cells via CRH-receptor 1-mediated activation of cAMP-PKA pathway. Endocrinology 143: 807–813 11861501 10.1210/endo.143.3.8694

[bib16] Griffith TS, Yu X, Herndon JM, Green DR, Ferguson TA (1996) CD95-induced apoptosis of lymphocytes in an immune privileged site induces immunological tolerance. Immunity 5: 7–16 8758890 10.1016/s1074-7613(00)80305-2

[bib17] Ito Y, Monden M, Takeda T, Eguchi H, Umeshita K, Nagano H, Nakamori S, Dono K, Sakon M, Nakamura M, Tsujimoto M, Nakahara M, Nakao K, Yokosaki Y, Matsuura N (2000) The status of Fas and Fas ligand expression can predict recurrence of hepatocellular carcinoma. Br J Cancer 82: 1211–1217 10735508 10.1054/bjoc.1999.1065 PMC2363358

[bib18] Kim YS, Kim KH, Choi JA, Lee JH, Kim HK, Won NH, Kim I (2000) Fas (APO-1/CD95) ligand and Fas expression in renal cell carcinomas: correlation with the prognostic factors. Arch Pathol Lab Med 124: 687–693 10782148 10.5858/2000-124-0687-FACLAF

[bib19] Makrigiannakis A, Zoumakis E, Kalantaridou S, Coutifaris C, Margioris AN, Coukos G, Rice KC, Gravanis A, Chrousos GP (2001) Corticotropin-releasing hormone promotes blastocyst implantation and early maternal tolerance. Nat Immunol 2: 1018–1024 11590404 10.1038/ni719

[bib20] Mastorakos G, Scopa CD, Vryonidou A, Friedman TC, Kattis D, Phenekos C, Merino MJ, Chrousos GP (1994) Presence of immunoreactive corticotropin-releasing hormone in normal and polycystic human ovaries. J Clin Endocrinol Metab 79: 1191–1197 7525629 10.1210/jcem.79.4.7525629

[bib21] Mastorakos G, Webster EL, Friedman TC, Chrousos GP (1993) Immunoreactive corticotropin-releasing hormone and its binding sites in the rat ovary. J Clin Invest 92: 961–968 8394389 10.1172/JCI116672 PMC294936

[bib22] Meng Y, Graves L, Do TV, So J, Fishman DA (2004) Upregulation of FasL by LPA on ovarian cancer cell surface leads to apoptosis of activated lymphocytes. Gynecol Oncol 95: 488–495 15581951 10.1016/j.ygyno.2004.07.052

[bib23] Mor G, Kohen F, Garcia-Velasco J, Nilsen J, Brown W, Song J, Naftolin F (2000) Regulation of fas ligand expression in breast cancer cells by estrogen: functional differences between estradiol and tamoxifen. J Steroid Biochem Mol Biol 73: 185–194 11070347 10.1016/s0960-0760(00)00081-9

[bib24] Mottolese M, Buglioni S, Bracalenti C, Cardarelli MA, Ciabocco L, Giannarelli D, Botti C, Natali PG, Concetti A, Venanzi FM (2000) Prognostic relevance of altered Fas (CD95)-system in human breast cancer. Int J Cancer 89: 127–132 10754489 10.1002/(sici)1097-0215(20000320)89:2<127::aid-ijc5>3.0.co;2-4

[bib25] Munakata S, Enomoto T, Tsujimoto M, Otsuki Y, Miwa H, Kanno H, Aozasa K (2000) Expressions of Fas ligand and other apoptosis-related genes and their prognostic significance in epithelial ovarian neoplasms. Br J Cancer 82: 1446–1452 10780525 10.1054/bjoc.1999.1073 PMC2363379

[bib26] Muramatsu Y, Sugino N, Suzuki T, Totsune K, Takahashi K, Tashiro A, Hongo M, Oki Y, Sasano H (2001) Urocortin and corticotropin-releasing factor receptor expression in normal cycling human ovaries. J Clin Endocrinol Metab 86: 1362–1369 11238533 10.1210/jcem.86.3.7299

[bib27] Niehans GA, Brunner T, Frizelle SP, Liston JC, Salerno CT, Knapp DJ, Green DR, Kratzke RA (1997) Human lung carcinomas express Fas ligand. Cancer Res 57: 1007–1012 9067260

[bib28] O'Connell J, Bennett MW, O'ullivan GC, Collins JK, Shanahan F (1999) The Fas counterattack: cancer as a site of immune privilege. Immunol Today 20: 46–52 10081230 10.1016/s0167-5699(98)01382-6

[bib29] O'Connell J, Houston A, Bennett MW, O'ullivan GC, Shanahan F (2001) Immune privilege or inflammation? Insights into the Fas ligand enigma. Nat Med 7: 271–274 11231613 10.1038/85395

[bib30] Okada K, Komuta K, Hashimoto S, Matsuzaki S, Kanematsu T, Koji T (2000) Frequency of apoptosis of tumor-infiltrating lymphocytes induced by fas counterattack in human colorectal carcinoma and its correlation with prognosis. Clin Cancer Res 6: 3560–3564 10999744

[bib31] Rabinowich H, Reichert TE, Kashii Y, Gastman BR, Bell MC, Whiteside TL (1998) Lymphocyte apoptosis induced by Fas ligand expressing ovarian carcinoma cells. Implications for altered expression of T cell receptor in tumor-associated lymphocytes. J Clin Invest 101: 2579–2588 9616229 10.1172/JCI1518 PMC508847

[bib32] Reimer T, Herrnring C, Koczan D, Richter D, Gerber B, Kabelitz D, Friese K, Thiesen HJ (2000) FasL:Fas ratio—a prognostic factor in breast carcinomas. Cancer Res 60: 822–828 10706087

[bib33] Remmele W, Hildebrand U, Hienz HA, Klein PJ, Vierbuchen M, Behnken LJ, Heicke B, Scheidt E (1986) Comparative histological, histochemical, immunohistochemical and biochemical studies on oestrogen receptors, lectin receptors, and Barr bodies in human breast cancer. Virchows Arch A Pathol Anat Histopathol 409: 127–147 2424168 10.1007/BF00708323

[bib34] Reubi JC, Waser B, Vale W, Rivier J (2003) Expression of CRF1 and CRF2 receptors in human cancers. J Clin Endocrinol Metab 88: 3312–3320 12843181 10.1210/jc.2002-021853

[bib35] Saas P, Walker PR, Hahne M, Quiquerez AL, Schnuriger V, Perrin G, French L, Van Meir EG, de Tribolet N, Tschopp J, Dietrich PY (1997) Fas ligand expression by astrocytoma *in vivo*: maintaining immune privilege in the brain? J Clin Invest 99: 1173–1178 9077524 10.1172/JCI119273 PMC507930

[bib36] Sato H, Nagashima Y, Chrousos GP, Ichihashi M, Funasak Y (2002) The expression of corticotropin-releasing hormone in melanoma. Pigment Cell Res 15: 98–103 11936276 10.1034/j.1600-0749.2002.1o063.x

[bib37] Shibakita M, Tachibana M, Dhar DK, Kotoh T, Kinugasa S, Kubota H, Masunaga R, Nagasue N (1999) Prognostic significance of Fas and Fas ligand expressions in human esophageal cancer. Clin Cancer Res 5: 2464–2469 10499620

[bib38] Suda T, Okazaki T, Naito Y, Yokota T, Arai N, Ozaki S, Nakao K, Nagata S (1995) Expression of the Fas ligand in cells of T cell lineage. J Immunol 154: 3806–3813 7706720

[bib39] Suda T, Tomori N, Yajima F, Odagiri E, Demura H, Shizume K (1986) Characterization of immunoreactive corticotropin and corticotropin-releasing factor in human adrenal and ovarian tumours. Acta Endocrinol (Copenh) 111: 546–552 3010623 10.1530/acta.0.1110546

[bib40] Tjuvajev J, Kolesnikov Y, Joshi R, Sherinski J, Koutcher L, Zhou Y, Matei C, Koutcher J, Kreek MJ, Blasberg R (1998) Anti-neoplastic properties of human corticotropin releasing factor: involvement of the nitric oxide pathway. *In vivo* 12: 1–10 9575420

[bib41] van Haaften-Day C, Russell P, Davies S, King NJ, Tattersall MH (2003) Expression of Fas and FasL in human serous ovarian epithelial tumors. Hum Pathol 34: 74–79 12605369 10.1053/hupa.2003.7

[bib42] Volm M, Koomagi R (2000) Relevance of proliferative and pro-apoptotic factors in non-small-cell lung cancer for patient survival. Br J Cancer 82: 1747–1754 10817513 10.1054/bjoc.1999.1210 PMC2374507

[bib43] Yakes FM, Wamil BD, Sun F, Yan HP, Carter CE, Hellerqvist CG (2000) CM101 treatment overrides tumor-induced immunoprivilege leading to apoptosis. Cancer Res 60: 5740–5746 11059768

[bib44] Zhang L, Conejo-Garcia JR, Katsaros D, Gimotty PA, Massobrio M, Regnani G, Makrigiannakis A, Gray H, Schlienger K, Liebman MN, Rubin SC, Coukos G (2003) Intratumoral T cells, recurrence, and survival in epithelial ovarian cancer. N Engl J Med 348: 203–213 12529460 10.1056/NEJMoa020177

[bib45] Zheng HC, Sun JM, Wei ZL, Yang XF, Zhang YC, Xin Y (2003) Expression of Fas ligand and caspase-3 contributes to formation of immune escape in gastric cancer. World J Gastroenterol 9: 1415–1420 12854132 10.3748/wjg.v9.i7.1415 PMC4615474

